# Notes and News

**Published:** 1899-07-01

**Authors:** 


### Notes and News.

(Collected and Communicated.)

### HOSPITAL MEETINGS.

Hospital for Epilepsy and Paralysis, Portland Terrace, Regent's Park.—Annual Meeting, Wednesday, July 5, three p.m.

Since the outbreak of small-pox at Hull 70 cases have been notified.

The receipts at the recent bazaar at the Albert Hall in aid of the funds of the Charing Cross Hospital have already amounted to upwards of £15,000, and more is expected to come in.

Since the outbreak of plague at Alexandria there have been upwards of thirty cases. At Hong Kong the number of cases still show an increase, though the epidemic is principally confined to one quarter of the town.

The University of Harvard is to have a College of Comparative Medicine. The fund given by Mr. Fabian has served to endow a chair of comparative pathology, and Mr. Pierce's bequests will endow a chair of comparative physiology and provide laboratories.

Yellow fever is epidemic again at Vera Cruz, in Mexico, and shows great virulence, the deaths during the first week in June being more than 60 per cent. of the cases reported. The Southern States of the Union seem to be taking things more calmly than they did last year, although the disease has already appeared in two places.

All grades and classes of railway servants will soon be provided with a convalescent home, the foundation of which has just been laid by Lord Amherst at Beltinge, near Herne Bay. The institution is due to the generosity of Mr. Passmore Edwards, who has provided for nearly the whole expense of establishing it by his gift of £6,000. The home will have about three and a half acres of ground attached, and will accommodate between fifty and sixty patients. The management will be representative of all the large railway companies.

Sir Henry Thompson and Sir Felix Semon have been elected honorary presidents of the International Medical Congress of 1900, which is to be held in Paris.

Doctors Roquette and Lapo have been experimenting with a serum for the cure of cancer at Brussels and in New Orleans, and when the results warrant it an announcement will be made to the Brussels Academy of Medicine.

The Liverpool School of Tropical Diseases is sending out its first expedition, headed by Major Ronald Ross, the director of the school. The purpose of the expedition is to discover the particular mosquito which disseminates malarial disease in Sierra Leone.

A garden party and fête given by the Ladies' Association in aid of the Tottenham Hospital is to be held on Saturday, July 1st, in the grounds of the hospital. The Countess of Portsmouth, who has kindly consented to reopen the Children's Ward, will receive purses towards the Maintenance Fund.

Dr. Hill, of the Bacteriological Laboratory of the Boston Health Department, has been examining a number of public telephones in that city. In several of the transmitters harmless microbes were found, as indeed was to be expected, but inoculation of guinea-pigs failed to show the presence of any pathogenic micro-organisms.

From a return that has been prepared in the statistical office of the London County Council it appears that the hospitals in London pay more than £20,000 a year in the form of rates to the various local authorities in the metropolis. It seems to us difficult to defend a system by which so large a sum subscribed for purely charitable purposes is yearly deducted as rates, while other buildings, such as places of worship, are allowed to go free.

At Sheffield, the schools where it is suspected that diphtheria is present are to be inspected by a medical man, the medical officer of health having reported the prevalence of diphtheria in the town, and his belief that the schools are the chief cause of the spread of the disease. Two female sanitary inspectors have been appointed. Their duties will be chiefly confined to the interior of dwelling-houses, and to reporting upon conditions of dirt and ill-feeding and ill-treatment of children.

In view of the tendency which has been so marked a feature in the plans of most of the hospitals constructed in this country during recent years, to spread the buildings over as large an area as possible and to avoid placing many storeys of wards one above the other, it is a matter of interest to find that the new annex to the St. Vincent's Hospital in New York, which was formally opened on May 28th, is seven storeys in height, and has on the top of it a spacious roof garden. It is said to be "fireproof" throughout, and we hope that this is something more than a mere form of speech. Many of the buildings which are commonly spoken of as of "fireproof construction" are in reality only "slow burners." Of course, such buildings are infinitely safer than those with the old-fashioned wooden floors, but something still better is required where it is a question of dealing with the sick and the bed-ridden.

